# Prof. Dr. med. Dr. h.c. mult. Gerd Meyer-Schwickerath inventor of light coagulation on his 100th birthday (July 10, 2020)

**DOI:** 10.1007/s00417-020-04819-0

**Published:** 2020-07-10

**Authors:** Bernd Kirchhof, Antonia Joussen, Norbert Bornfeld, Achim Wessing

**Affiliations:** 1 Department of Ophthalmology, University of Cologne, Cologne, Germany; 2 Charite Berlin, Berlin, Germany; 3 University of Duesburg-Essen, Duesburg, Germany

**Fig. 1:**
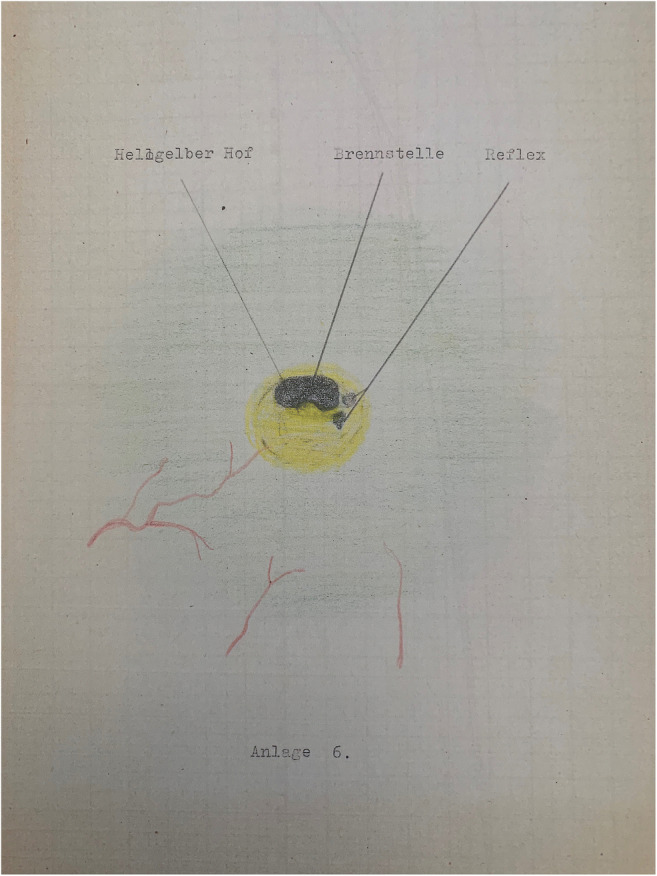
The 1947 drawing of a retinopathia solaris that sparked Meyer- Schwickerath’s idea of photocoagulation. Courtesy of Rolf Meyer-Schwickerath

People become inspired. But it often takes years of tinkering to develop an idea for use. It also requires the ability to integrate external expertise, as necessary, and to learn from failures. The drawing of a “retinopathia solaris” shown in Fig. 1 gave Gerd Meyer-Schwickerath in 1946 the idea of therapeutically focusing light on the retina. For the first time, the goal was to prevent rhegmatogenous retinal detachment. Transpupillary, the retina can be reached more directly than by trans-scleral diathermy that was common at the time. The illustration shown is taken from a doctoral thesis that Meyer-Schwickerath co-supervised under Oswald Marchesani in Hamburg [1]. The injury was caused by gazing into the sun during the eclipse of July 9, 1945. It is known that the arc lamp is not bright enough for the small pupil. Sunlight is sufficient but not reliably available. Only a collaboration with Hans Littmann (Carl-Zeiss Oberkochen) brought about the breakthrough with the xenon light source. On August 22, 1949, Meyer-Schwickerath carried out the first light coagulation on a patient with imminent retinal detachment. Abnormal vessels and small tumors could be obliterated in the same way. Intraocular tumors were particularly disastrous as they appear as retinoblastomas in the first few months of life or could endanger eyesight and life as malignant melanomas later in life.

Based on the therapeutic possibilities of light coagulation, Meyer- Schwickerath was one of the first to set up a specialized center for treatment of these tumors and able to significantly improve the chances for children and adults. Finally, there was yet another major tragedy in ophthalmology: juvenile diabetic retinopathy, predictable but not treatable (Duke-Elder 1967). Meyer-Schwickerath learned that neovascularization could only be effectively suppressed when he coagulated the retina in a disseminated manner and did not only target the abnormal vessels. In this regard, Meyer-Schwickerath quoted the Heinrich-Heine verse: “At first I wanted to despair, and I thought I would never wear it. Later I wore it, but just don't ask me how.” A prospective, randomized study of light coagulation in diabetic retinopathy needed to be stopped after evaluating the interim results. The effect was so resounding that it seemed unethical to the control group to withhold light coagulation. This accolade assisted light coagulation of diabetic retinopathy to a breakthrough. Anyone who looks at their own fundus photos and experiences for themselves how optic disc proliferations permanently disappear with few, but strong peripheral light or laser scars will understand the appreciation of light coagulation to the present day.

More than awards and medals, Meyer-Schwickerath valued the gratitude that his patients showed him. That was—as he said to himself—his highest reward. He fulfilled his duties as a teacher, host, pioneer, and full professor with wit and without "great fuss" about himself. Among the things that people most regret in their last hours, a brainchild is often mentioned, from which the pursuit is somehow distracted. Gerd Meyer-Schwickerath realized his idea. We all benefit from him.
